# Supplementary material for: Synthesis of Thymidine Phosphorylase Inhibitor Based on Quinoxaline Derivatives and Their Molecular Docking Study
Source: Molecules. 2019 Mar 13;24(6):1002. doi: 10.3390/molecules24061002 (PMC6471342; doi:10.3390/molecules24061002)
Supplement: Supplementary file 1 [file molecules-24-01002-s001.pdf]

S.No.	Structure	IC$_{50}$ (μM ± SEM[a])	SMILES
**1**	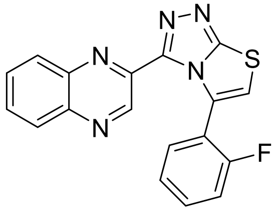	13.60 ± 0.4	**FC1=CC=CC=C1C5=CSC4=NN=C(N45)C3=NC2=CC=CC=C2N=C3**
**2**	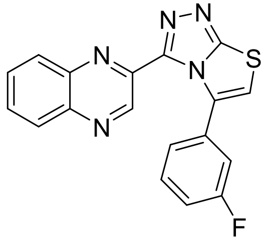	26.10 ± 0.70	**FC1=CC(C5=CSC4=NN=C(N45)C3=NC2=CC=CC=C2N=C3)=CC=C1**

**3**	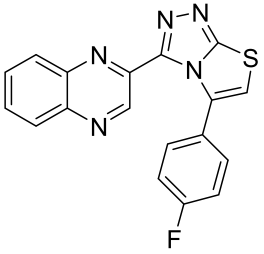	18.10 ± 0.50	**FC(C=C5)=CC=C5C4=CSC3=NN=C(N34)C2=NC1=CC=CC=C1N=C2**
**4**	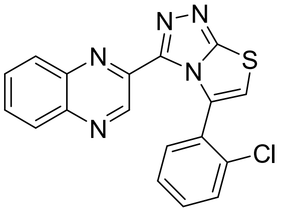	27.40 ± 0.60	**ClC1=CC=CC=C1C5=CSC4=NN=C(N45)C3=NC2=CC=CC=C2N=C3**
**5**	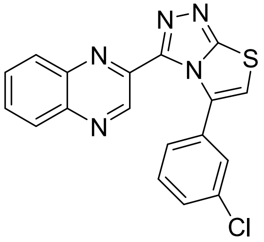	33.40 ± 0.80	**ClC1=CC(C5=CSC4=NN=C(N45)C3=NC2=CC=CC=C2N=C3)=CC=C1**

**6**	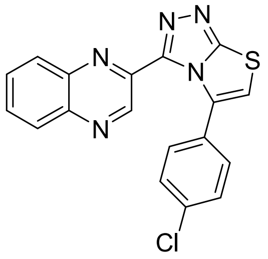	24.40 ± 0.60	**ClC(C=C5)=CC=C5C4=CSC3=NN=C(N34)C2=NC1=CC=CC=C1N=C2**
**7**	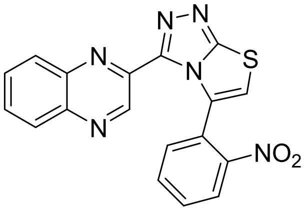	34.70 ± 0.80	**O=[N+](C1=CC=CC=C1C5=CSC4=NN=C(N45)C3=NC2=CC=CC=C2N=C3)[O-]**
**8**	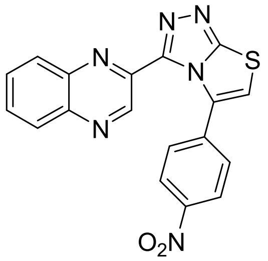	47.50 ± 0.90	**O=[N+](C(C=C5)=CC=C5C4=CSC3=NN=C(N34)C2=NC1=CC=CC=C1N=C2)[O-]**

**9**	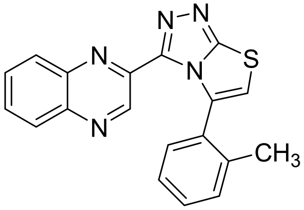	56.40 ± 1.20	**CC1=CC=CC=C1C5=CSC4=NN=C(N45)C3=NC2=CC=CC=C2N=C3**
**10**	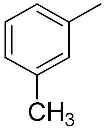	N. A.	**CC1=CC(C5=CSC4=NN=C(N45)C3=NC2=CC=CC=C2N=C3)=CC=C1**
**11**	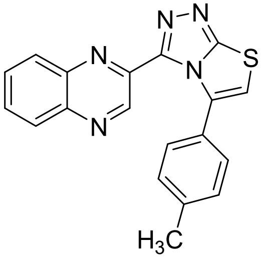	N. A.	**CC(C=C5)=CC=C5C4=CSC3=NN=C(N34)C2=NC1=CC=CC=C1N=C2**
**12**	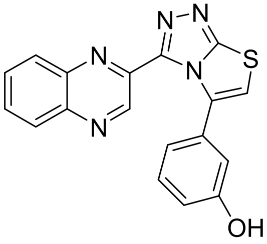	33.20 ± 0.75	**OC1=CC(C5=CSC4=NN=C(N45)C3=NC2=CC=CC=C2N=C3)=CC=C1**

**13**	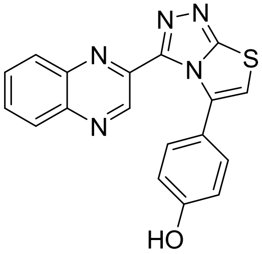	18.30 ± 0.55	**OC(C=C5)=CC=C5C4=CSC3=NN=C(N34)C2=NC1=CC=CC=C1N=C2-**
**14**	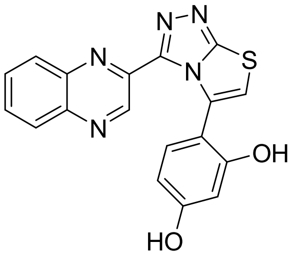	13.20 ± 0.40	**OC(C=C5O)=CC=C5C4=CSC3=NN=C(N34)C2=NC1=CC=CC=C1N=C2**
**15**	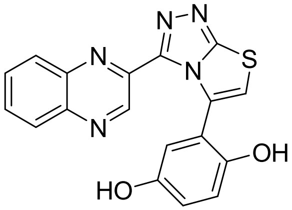	15.20 ± 0.50	**OC1=CC=C(O)C=C1C5=CSC4=NN=C(N45)C3=NC2=CC=CC=C2N=C3**

**16**	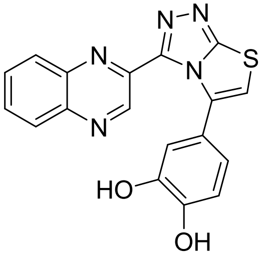	3.50 ± 0.20	**OC1=C(O)C=CC(C5=CSC4=NN=C(N45)C3=NC2=CC=CC=C2N=C3)=C1**
**17**	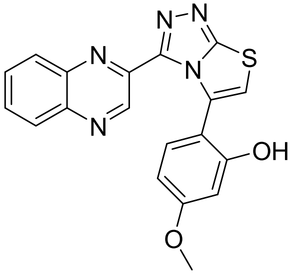	24.20 ± 0.70	**OC1=CC(OC)=CC=C1C5=CSC4=NN=C(N45)C3=NC2=CC=CC=C2N=C3**
**18**	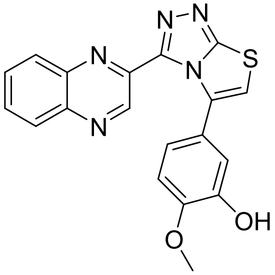	16.90 ± 0.60	**COC(C(O)=C5)=CC=C5C4=CSC3=NN=C(N34)C2=NC1=CC=CC=C1N=C2**

**19**	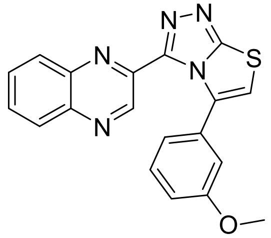	N. A.	**COC1=CC(C5=CSC4=NN=C(N45)C3=NC2=CC=CC=C2N=C3)=CC=C1**
**20**	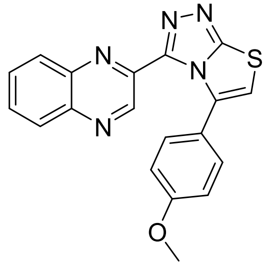	N. A.	**COC(C=C5)=CC=C5C4=CSC3=NN=C(N34)C2=NC1=CC=CC=C1N=C2**
**21**	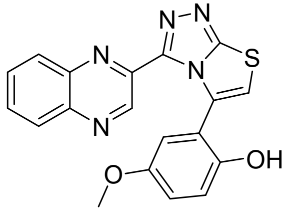	26.20 ± 0.50	**OC1=CC=C(OC)C=C1C5=CSC4=NN=C(N45)C3=NC2=CC=CC=C2N=C3**

**22**	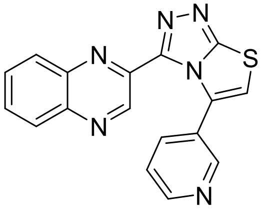	N. A.	**C12=CC=CC=C1N=C(C3=NN=C4N3C(C5=CC=CN=C5)=CS4)C=N2**
**23**	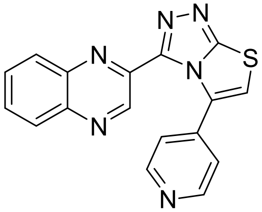	N. A.	**C12=CC=CC=C1N=C(C3=NN=C4N3C(C5=CC=NC=C5)=CS4)C=N2**
**24**	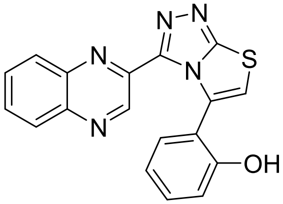	13.10 ± 0.30	**OC1=CC=CC=C1C5=CSC4=NN=C(N45)C3=NC2=CC=CC=C2N=C3**

**25**	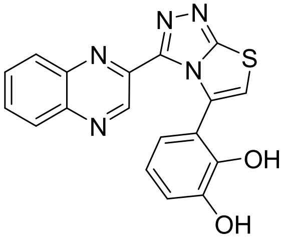	3.20 ± 0.10	**OC1=C(O)C=CC=C1C5=CSC4=NN=C(N45)C3=NC2=CC=CC=C2N=C3**
**Std Drug**	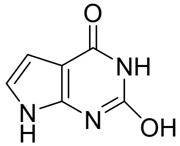	**38.68 ± 4.42**	**OC(N1)=NC2=C(C=CN2)C1=O**

N.A = not Active; Sem[a] = Standard error Mean
